# Structural basis of Zika virus helicase in recognizing its substrates

**DOI:** 10.1007/s13238-016-0293-2

**Published:** 2016-07-18

**Authors:** Hongliang Tian, Xiaoyun Ji, Xiaoyun Yang, Zhongxin Zhang, Zuokun Lu, Kailin Yang, Cheng Chen, Qi Zhao, Heng Chi, Zhongyu Mu, Wei Xie, Zefang Wang, Huiqiang Lou, Haitao Yang, Zihe Rao

**Affiliations:** 1 School of Life Sciences, Tianjin University, Tianjin, 300072 China; 2 Tianjin International Joint Academy of Biotechnology and Medicine, Tianjin, 300457 China; 3 The State Key Laboratory of Pharmaceutical Biotechnology, School of Life Sciences, Nanjing University, Nanjing, 210023 China; 4 State Key Laboratory of Agro-Biotechnology, College of Biological Sciences, China Agricultural University, Beijing, 100193 China; 5 College of Life Sciences, Nankai University, Tianjin, 300071 China; 6 Cleveland Clinic Lerner College of Medicine of Case Western Reserve University, Cleveland, OH 44195 USA; 7 Department of Molecular Biophysics and Biochemistry, Yale University, New Haven, CT 06520 USA

**Keywords:** Zika virus, helicase, ATP, crystal structure, flavivirus

## Abstract

The recent explosive outbreak of Zika virus (ZIKV) infection has been reported in South and Central America and the Caribbean. Neonatal microcephaly associated with ZIKV infection has already caused a public health emergency of international concern. No specific vaccines or drugs are currently available to treat ZIKV infection. The ZIKV helicase, which plays a pivotal role in viral RNA replication, is an attractive target for therapy. We determined the crystal structures of ZIKV helicase-ATP-Mn^2+^ and ZIKV helicase-RNA. This is the first structure of any flavivirus helicase bound to ATP. Comparisons with related flavivirus helicases have shown that although the critical P-loop in the active site has variable conformations among different species, it adopts an identical mode to recognize ATP/Mn^2+^. The structure of ZIKV helicase-RNA has revealed that upon RNA binding, rotations of the motor domains can cause significant conformational changes. Strikingly, although ZIKV and dengue virus (DENV) apo-helicases share conserved residues for RNA binding, their different manners of motor domain rotations result in distinct individual modes for RNA recognition. It suggests that flavivirus helicases could have evolved a conserved engine to convert chemical energy from nucleoside triphosphate to mechanical energy for RNA unwinding, but different motor domain rotations result in variable RNA recognition modes to adapt to individual viral replication.

**Electronic supplementary material:**

The online version of this article (doi:10.1007/s13238-016-0293-2) contains supplementary material, which is available to authorized users.

## Introduction

Zika virus (ZIKV) belongs to the *Flavivirus* genus which contains important human pathogens such as dengue (DENV), yellow fever (YFV), West Nile (WNV), Japanese encephalitis (JEV) and tick-borne encephalitis (TBEV) viruses (Pierson and Diamond, 2013). ZIKV was first isolated in 1947 from a febrile sentinel rhesus monkey in the Zika forest of Uganda (Wikan and Smith, 2016). As an arthropod-borne flavivirus, ZIKV is transmitted by multiple *Aedes* mosquitoes (Dick et al., 1952). Typically, human infection by ZIKV caused a mild and self-limiting illness, characterized with fever, headache, arthralgia, myalgia, and maculopapular rash (Ioos et al., 2014). In April 2007, a large epidemic of Asian genotype ZIKV broke out in Yap Island and Guam, Micronesia, bringing ZIKV to global attention (Duffy et al., 2009; Haddow et al., 2012). From 2013 to 2014, the Asian genotype was also confirmed as the culprit for numerous epidemics among several Pacific Islands, including French Polynesia, New Caledonia, Cook Islands, Tahiti, and Easter Island (Lazear and Diamond, 2016). In 2015, widespread ZIKV infection was reported in Brazil and other parts of South America, with an estimated case counts of 1.3 million cases (Hennessey et al., 2016; Mlakar et al., 2016). Recent studies showed that ZIKV was identified in fetal brain tissue, presumably accounting for the sharp increase of congenital microcephaly in the epidemic areas (Brasil et al., 2016; Mlakar et al., 2016; Rodrigues, 2016). Upon ZIKV infection, significant cellular death of neural stem cells was shown to be responsible for the inhibitory role of ZIKV on fetal brain development (Tang et al., 2016). However, no effective vaccines or therapies are currently available to prevent or treat ZIKV infection. With the increasing case numbers and potential risk of global spread, ZIKV is becoming a great challenge to the public health of the Western Hemisphere as well as the whole world (Lazear and Diamond, 2016).

The genome of ZIKV is composed of a positive-sense single strand RNA. Viral replication begins with the translation of its RNA genome into a large polypeptide, which is then proteolytically cleaved into 3 structural proteins (C, prM/M, and E), and 7 non-structural proteins (NS1, NS2A, NS2B, NS3, NS4A, NS4B, and NS5) (Pierson and Diamond, 2013). The NS3 protein plays an essential role in viral polypeptide processing and genomic replication, with a protease domain at its N-terminus and a helicase domain at the C-terminus. Upon RNA binding, the helicase domain exhibits intrinsic nucleoside triphosphatase activity, which then provides the chemical energy to unwind viral RNA replication intermediates to facilitate replication of the viral genome together with RNA-dependent RNA polymerase (NS5) (Lindenbach and Rice, 2001). Given its essential role in genome replication, ZIKV helicase could be an attractive target for drug development against ZIKV (Noble et al., 2010). Recently, we have reported the apo-helicase of ZIKV (Tian et al., 2016), but the mechanisms of how ZIKV helicase recognizes nucleoside triphosphate and viral RNA is still largely unknown, hindering the development of antiviral drugs. Here we report the crystal structures of ZIKV helicase-ATP-Mn^2+^ and ZIKV helicase-RNA, which help elucidate how ZIKV recognizes its substrates during replication and provide structural insight for rational drug design.

## Results and discussion

### ATP hydrolysis and RNA unwinding assays

**Figure 1:**
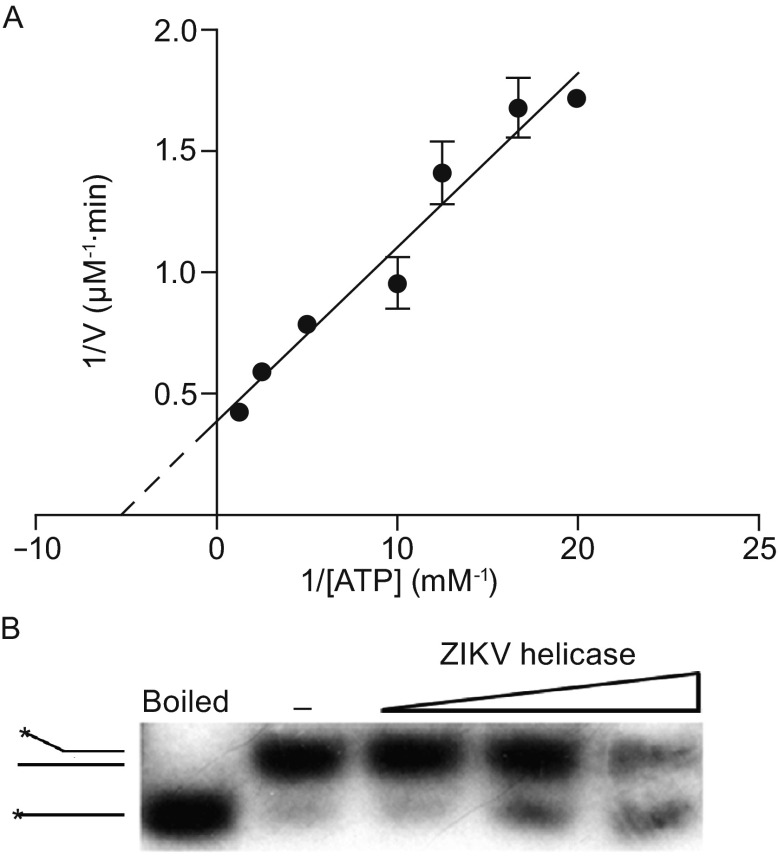
**The ATPase and RNA unwinding activities of ZIKV helicase**. (A) Determination of ATP hydrolysis activity of ZIKV helicase. The ATPase assay was carried out with 20 nmol/L of enzyme in the presence of the indicated concentrations of ATP for 20 min at 25°C. The double-reciprocal plot was fitted according to the Michaelis-Menten equation. (B) Measurement of dsRNA unwinding activity of ZIKV helicase. RNA unwinding activity of ZIKV helicase was assayed using a radiolabeled dsRNA substrate. The first lane is the positive control (heat-denatured duplex) and the second lane is the negative control (without ZIKV helicase)

Flavivirus helicases have both ATP hydrolysis and RNA unwinding activities. For structural studies, we have made two constructs (helicase_172–617_ and helicase_180–617_) to express the ZIKV helicase. The kinetic parameters of ATP hydrolysis for the long form of the ZIKV helicase_172–617_ were determined using the Malachite green assay as reported previously (Lanzetta et al., 1979). The resulting data show that ZIKV helicase displays ATPase activity with *K*_*m*_ = 191 ± 26 μmol/L and *k*_*cat*_ = 2.3 ± 0.1 s^−1^ (Fig. 1A). The short form of the ZIKV helicase_180–617_ can also hydrolyze ATP and the activity difference between the short form and the long form are negligible. The RNA unwinding activity was assayed using radiolabeled double-stranded (ds) RNA, in the presence of Mg^2+^, ATP, and various concentrations of enzyme (Fig. 1B). It demonstrated that ZIKV helicase displayed strand displacement activity for dsRNA as other flavivirus helicases.

### Structure determination

To elucidate the molecular mechanisms of ZIKV helicase in recognizing ATP/Mn^2+^ and RNA, we determined the crystal structures of ZIKV helicase_180–617_ complexed with ATP/Mn^2+^ and ZIKV helicase_172–617_ complexed with a 7-mer RNA (5′-AGAUCAA-3′) at 2.2 Å and 1.7 Å, respectively (Table S1).

### The structure of the ZIKV helicase in complex with ATP and Mn^2+^

**Figure 2:**
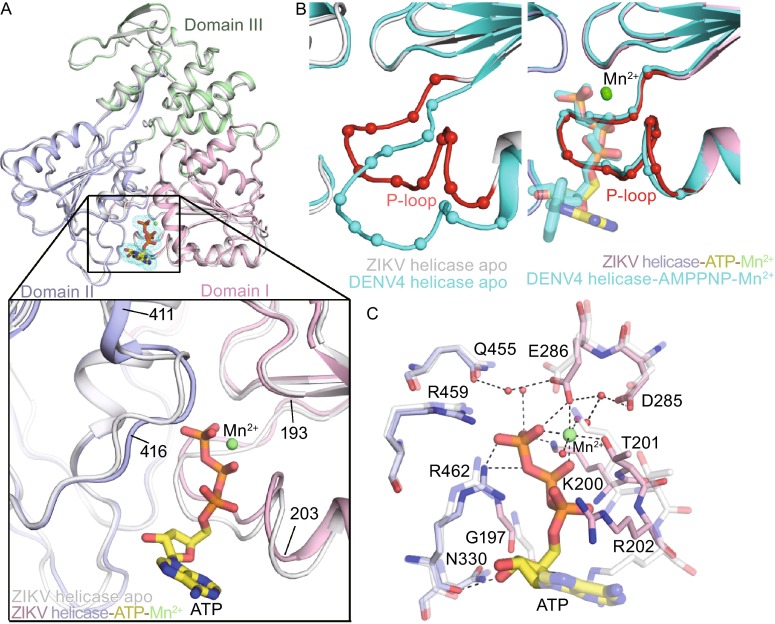
**Structure of the ZIKV helicase in complex with ATP/Mn** ^**2+**^. (A) Overall fold of ZIKV helicases, with cartoon representation of apo form (white) overlaid to the complex (the three domains are colored respectively). The ATP is drawn as sticks and mesh; Mn^2+^ as green sphere. A detailed comparison for the ATP binding sites of the two structures is depicted in the zoomed view below. (B) A close-up view of the NTPase active site. P-loops are represented by superimposition of the structures of ZIKV (white, with the P-loop highlighted in red) and DENV4 (cyan) apo-helicases in the left panel and their complexes in the right panel. The DENV4 helicase complex was bound to AMPPNP and Mn^2+^ (PDB code 2JLR). (C) Interactions at NTPase active site by superposition of the ZIKV helicase complexed with ATP and Mn^2+^ (solid) with its apo enzyme (semitransparent, PDB code 5JMT)

Due to the nucleotide hydrolysis activity, there is no structure reported for any flavivirus helicase complexed with ATP. Instead, the nucleotide analog 5′-adenylyl-β, γ-imidodiphosphate (AMPPNP) has been used to study helicase-nucleotide interaction (Luo et al., 2008). Fortunately, we captured the ZIKV helicase_180–617_ in an ATP-bound state, which is the first structure of any flavivirus helicase bound to ATP, even though it displays NTPase activity. The overall structure of the ZIKV helicase_180–617_ in complex with ATP/Mn^2+^ is similar to that of its apo-form (overall RMSD 0.557 Å), except for the movement of the P-loop (residues 193–203) towards the inner core and lateral movement of main-chain residues 411–416 to better accommodate ATP (Fig. 1A and 1B). As we have reported previously, the P-loop, which is critical for NTP binding and catalysis, has a variety of structural conformations among flavivirus apo-helicases (Tian et al., 2016). It is worthwhile to note that upon nucleotide binding, ATP/Mn^2+^ induced marked conformational change of the P-loop, also seen in DENV4 helicase (Luo et al., 2008) (Fig. 2B). Interestingly, we found that the P-loop and other elements which constitute the NTP binding pockets of ZIKV and DENV4 helicases undergo different local rearrangements, but then adopt an identical mode to recognize ATP/Mn^2+^. However, their apo-conformations are distinct from each other. This suggests that flavivirus helicases have evolved a conserved molecular engine to convert chemical energy into mechanical energy for unwinding viral RNA during replication.

In the ZIKV helicase_180–617_-ATP-Mn^2+^ tertiary structure, ATP/Mn^2+^ are located at the cleft between Domain I and II (Fig. 2A and 2C). Substrate binding causes an inward reorientation of side-chain of K200 to stabilize the triphosphate moiety of ATP and a flipping of the side-chain of R202 towards the solvent to leave room for sugar moiety. The triphosphate moiety of ATP adopts an extended conformation as seen in DENV4 helicase in complex with AMPPNP (Luo et al., 2008). The Mn^2+^ ion is coordinated in an octahedral geometry by side chains of E286 (motif II) and T201, two ordered water molecules and two oxygen atoms from the β/γ phosphate groups of the ATP molecule, which stabilize the nucleoside triphosphate. The ATP molecule makes additional contacts with G197, K200, R202 (P-loop), R459, R462 (motif VI) and other ordered water molecules (Fig. 2C). Among them, K200 is responsible for interacting with the γ-phosphate of the nucleotide during transition state. The 3′-OH group of the ribose forms hydrogen bonds with the carbonyl oxygen of R462 and the side chain amide group of N330. The ribose group of ATP bulges out from the binding pocket and no clear electron densities are observed for the adenine group, suggesting that the ZIKV helicase may not have nucleotide specificity for its NTPase activity.

### The structure of ZIKV helicase in complex with RNA

#### Overall structure

**Figure 3:**
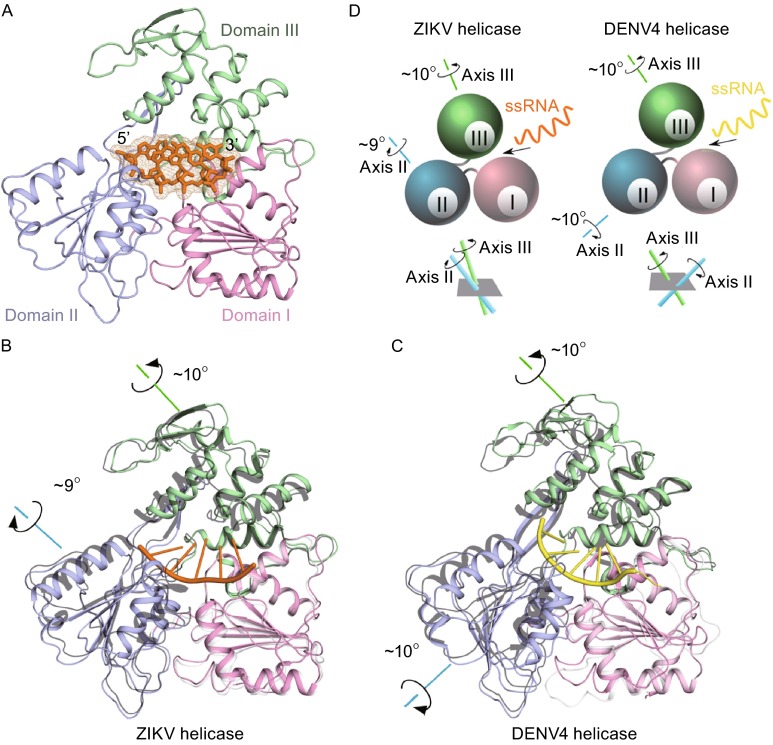
**Structure of the ZIKV helicase in complex with RNA**. (A) Cartoon representation of overall fold of the ZIKV helicase-RNA complex with three domains colored and marked respectively. The ssRNA is shown in orange sticks and meshes. (B) Overlay of the ZIKV helicase-RNA complex structure and its apo form (grey). The RNA is shown in orange. (C) Overlay of the DENV4 helicase-RNA complex structure and its apo form (grey). The RNA is shown in yellow. The rotations of domain II and III upon RNA binding are depicted accordingly. (D) Schematic illustration of the different modes of domain rotations for ZIKV and DENV4 helicases upon RNA binding

In the structure of the ZIKV helicase_172–617_ in complex with a 7-mer RNA, the single-stranded (ss) RNA runs through Domain II to Domain I in an extended conformation with the bases stacked against each other, separating these two domains from Domain III. The 3′ end of ssRNA binds to Domain I, while the 5′ end mainly interacts with Domain II. Nucleotides 1–5 are well ordered and the electron densities are mostly invisible for nucleotides 6–7 (Fig. 3A).

#### Conformational changes upon RNA binding

Compared with its apo-form, the ZIKV helicase undergoes obvious conformational changes, largely due to a rotation of Domain II and Domain III, once it binds to ssRNA. Domain II rotates about 9° away from Domain I in a rigid-body rotation mode along axis II in the direction as noted in Fig. 3B and 3D. However, Domain III rotates about 9° away from Domain I in the opposite direction along axis III, which is approximately parallel to axis II (Fig. 3B and 3D). This rotor domain rotation caused two α-helices (residues 365–379, and residues 390–400) in Domain II and two α-helices (residues 525–537, and residues 602–615) in Domain III to move away from the RNA binding groove in an opposite direction, enlarging the groove to accommodate the ssRNA. This natural design functions like a double-leaf swing gate with each leaf opening in a reverse direction to the other (Fig. 3B and 3D). Interestingly, the motor domain rotation mode in the ZIKV helicase is distinct from that in the DENV4 helicase structure (Luo et al., 2008). In the DENV4 helicase, the rotation axis for Domain II, however, is almost vertical to that for Domain III, but the rotation directions are identical (Fig. 3C and 3D).

#### RNA recognition

**Figure 4:**
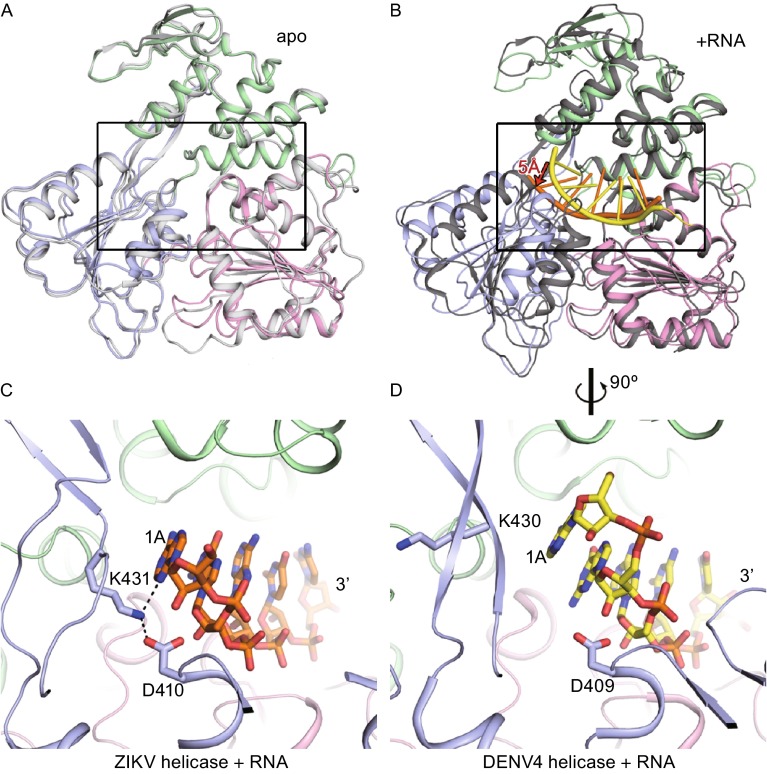
**RNA recognition modes for ZIKV and DENV4 helicases**. (A) Superposition of ZIKV (domains colored respectively) and DENV4 (white) apo-helicases. (B) Superposition of ZIKV (domains colored respectively and RNA in orange) and DENV4 (black and RNA in yellow) helicase-RNA complex. The distance between the sugar groups of nucleotide 1 in ZIKV and DENV4 helicases is marked in red. (C and D) show the conformation of subsite 1 of the ZIKV helicase-RNA complex (C) and the DENV4 helicase-RNA complex (D) in a 90° rotated view of (B). RNAs are shown in sticks and proteins are shown in ribbon with domains colored differently

**Figure 5:**
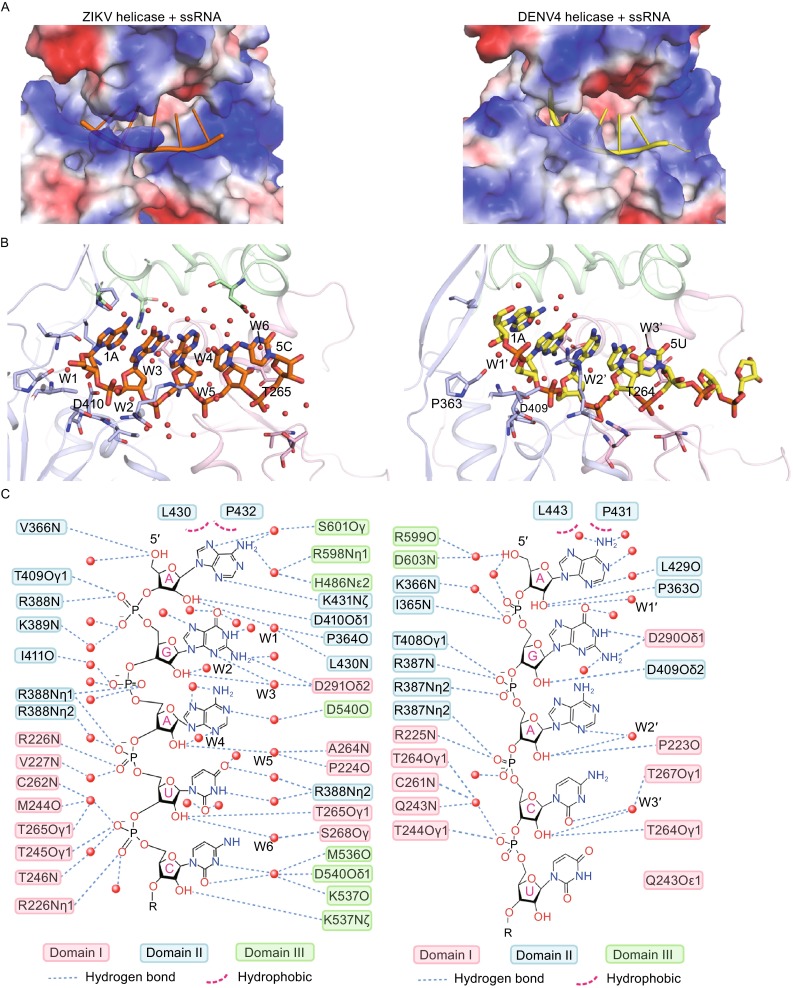
**Comparison of protein-RNA interactions for ZIKV and DENV4 helicases**. Left panels are for ZIKV helicase-RNA complex and right panels for DENV4. (A) The electrostatic surface representations showing the tunnel for RNA binding. Positive potentials are colored blue and the negative are colored red. The nucleic acids are shown in orange (ZIKV helicase) and yellow (DENV4 helicase). (B) Interactions in RNA binding tunnels. Proteins are shown in ribbon and colored according to domains. RNAs and interacting residues from the helicases are shown in sticks. Water molecules are shown in red spheres. (C) Detailed view for the protein-ssRNA interactions

At first glance, the residues for RNA binding are well conserved in both the ZIKV and DENV apo-helicases (Fig. 4A). Additionally, similar to the DENV helicase, the ZIKV helicase binds to ssRNA by a positively charged tunnel identified along the domain boundary of Domain III, which directly interacts with Domain I and Domain II as well (Figs. 3–5). However, to our surprise, the exact RNA recognition mode differs markedly between these two structures due to the distinct motor domain rotation upon ssRNA binding. Compared with the structure of the DENV4 helicase complexed with a 12-mer RNA (Luo et al., 2008), the sugar-phosphate backbone of nucleotides 1–3 is more extended in the ZIKV helicase. The sugar group of nucleotide 1 (A) in the ZIKV helicase is ~5 Å away from that in the DENV4 helicase (Fig. 4B). This causes different conformations of subsite 1 in the ZIKV and DENV4 helicases to better fit the adenine. In particular, the side chain of K431 in the ZIKV helicase points to the inner core, forming a salt bridge with the side chain of D410 and a weak hydrogen bond with N3 atom of the adenine base (Fig. 4C). The corresponding residue (K430) in the DENV4 helicase, however, projects its side chain towards the solvent (Fig. 4D). In addition, as seen in both the ZIKV and DENV4 helicase structures, a complex network of water molecules is important for ssRNA binding, yet these water molecules may play different roles in recognizing an individual nucleotide. The specificity of the ZIKV helicase for RNA relies on multiple hydrogen bonds between the 2′-OH moieties from the ssRNA and the carbonyl oxygen of D410, side chain oxygen of T265, and six water molecules (W1–6) (Fig. 5B and 5C). In the DENV4 helicase, however, P363, P223, D409, T264 and the other three water molecules are responsible for interacting with 2′-OH moieties in the RNA, suggesting that the ZIKV helicase might depend more on the water network in discriminating between RNA and DNA than the DENV4 helicase (Fig. 5B and 5C). The detailed difference between ZIKV and DENV4 helicases for RNA interaction is shown in Fig. 5C.

Because of its essential role for replication, a viral helicase is an attractive target whose accurate mechanism is still largely unknown. Flavivirus helicases possess nucleoside triphosphatase activity, which enables the enzyme to convert chemical energy to unwind viral RNA replication intermediates. Our structures presented here can help deepen our understanding of this process and provide structural basis for rational drug design. Interestingly, although there exists conformational variety in the NTP binding pocket of apo-helicases among different flaviviruses, they undergo conformational changes to adopt an identical mode to bind NTPs, which may result from the natural selection for the same function of hydrolyzing NTPs. On the other hand, to our surprise, although the residues are well conserved for RNA binding between different flavivirus apo-helicases, distinct rotations of motor domains would cause different manners to recognize their individual RNAs during replication. These findings suggest that flaviviruses could have evolved a conserved engine to convert chemical energy to mechanical energy, but variable RNA recognition modes to adapt to their individual replication.

## Materials and methods

### Cloning and expression

The cloning and expression of the ZIKV helicase_172–617_ (residues 172 to 617) has been described previously (Tian et al., 2016). The short form of catalytic domain_180–617_ (residues 180 to 617) was amplified by PCR using the forward primer 5′-CGCGGATCCGAGCCGTCAATGTTGAAG-3′ and the reverse primer 5′-CCGCTCGAGTTACCGTTTTCCGGCTGCGAA-3′. The underlined regions correspond to *Bam*HI and *Xho*I sites, respectively. The coding sequence for helicase_180–617_ was cloned into the vector pET.32M.3C and fused at its N-terminus to thioredoxin and a (His)_6_ tag followed by PreScission Protease (GE) cleavage site. Transformed *Escherichia coli* BL21 (DE3) clones were grown in LB medium at 37°C and then induced by 0.2 mmol/L isopropyl-β-D-thiogalactopyranoside at 16°C. After overnight growth, cells were harvested via centrifugation.

### Protein purification

The purification of ZIKV helicase_172–617_ has been described previously (Tian et al., 2016). Briefly, the Trx-(His)_6_-helicase_172–617_ was purified by Ni Sepharose (GE) affinity chromatography and cleaved with PreScission Protease, followed by anion-exchange chromatography and size exclusion chromatography. The purification of ZIKV helicase_180–617_ was described below. Cells resuspended in lysis buffer A (20 mmol/L Na_2_HPO_4_, pH 8.0, 0.5 mol/L NaCl and 20 mmol/L imidazole) were lysed by high pressure homogenization and the lysate was clarified by centrifugation at 30,000 ×*g* for 40 min at 4°C. The supernatant was purified by Ni Sepharose (GE) affinity chromatography equilibrated with buffer A. Proteins were eluted using buffer A supplemented with 250 mmol/L imidazole. After concentration by ultrafiltration and dilution in buffer B (20 mmol/L Na_2_HPO_4_, pH 8.0, 0.5 mol/L NaCl), the fraction containing Trx-(His)_6_-ZIKV helicase_180–617_ was cleaved with PreScission Protease at 4°C for approximately 12 h. The cleavage mixture of ZIKV helicase_180–617_ was loaded onto a HiTrap S 5 mL column (GE) pre-equilibrated with buffer C (50 mmol/L HEPES, pH 7.0, 50 mmol/L NaCl and 5% glycerol) and also eluted using a linear NaCl concentration gradient. The concentrated proteins of interest were subjected to a final gel-filtration purification step through a HiLoad 16/600 Superdex 200^TM^ PG column (GE) in buffer D (10 mmol/L Tris-HCl, pH 8.0, 150 mmol/L NaCl, 5 mmol/L dithiothreitol and 5% glycerol).

### ATPase activity assay

The ATP activity assay was carried out using the QuantiChromTM ATPase/GTPase Assay Kit (BioAssay Systems). The ZIKV helicase_172–617_ was preincubated at a concentration of 20 nmol/L in 20 μL assay buffer (40 mmol/L Tris, 80 mmol/L NaCl, 8 mmol/L MgAc_2_, 1 mmol/L EDTA, pH 7.5) in a 96-well plate. The reaction was carried out with 10 μL ATP at various concentrations for 20 min at 25°C and then terminated by adding 200 μL of reagent buffer. Followed by incubation with reagent buffer for 30 min at the room temperature, the absorbance was measured at 620 nm. The *K*_*m*_ and *k*_*cat*_ of the enzyme were obtained from a double-reciprocal plot with the GraphPad Prism Software.

### RNA unwinding

To obtain the partial dsRNA substrate (11-nucleotide complementation) for helicase unwinding activity assay, the R1 (5′-AGCCUAAAUUUCAAUCCCG-3′) strand was labeled by using [γ-^32^P]ATP (3000 Ci/mmol, Perkin-Elmer) and T4 polynucleotide kinase (Thermo scientific) for 1 h at 37°C. After ethanol precipitation, the labeled R1 was annealed with the R2 (5′-CGGGAUUGAAAGGACUUAC-3′) strand by heating to 100°C in annealing buffer (10 mmol/L Tris-Cl, pH 7.5, 100 mmol/L NaCl, 1 mmol/L EDTA) and cooled down slowly to room temperature. The annealed duplex was purified by a 15% native polyacrylamide gel electrophoresis in TBE buffer (45 mmol/L Tris, 45 mmol/L boric acid, 2 mmol/L EDTA, pH 8.0) and dissolved in TE buffer to yield a 10 nmol/L substrate.

The assay was performed using 20 μL reaction mixture containing 50 mmol/L HEPES (pH 7.5), 50 mmol/L NaCl, 2.5 mmol/L MgCl_2_, 10 mmol/L ATP, 5 U RNase inhibitor (New England Biolabs), 0.5 nmol/L of RNA substrate and the ZIKV helicase_172–617_ at various concentrations or an equivalent volume of the protein storage buffer (negative control). The mixtures were incubated for 30 min at 30°C and the reactions were terminated by adding 5 μL-loading dye (0.25 mol/L EDTA, 0.5% SDS, 50% glycerol, 0.01% bromophenol blue) to the mixtures. The boil mixture (without helicase) was boiled in boiling water for 3 min then chilled on ice quickly. All these mixtures were subjected to electrophoresis using a 12% native polyacrylamide gel. The radioisotopic substrates were detected by X-OMAT BT Film (Carestream).

### Crystallization

Crystals for the ATP (Sangon Biotech) complex were obtained by cocrystallization of the ZIKV helicase_180–617_ at a concentration of 5 mg/mL, with 5 mmol/L MnCl_2_ and 5 mmol/L ATP in 0.1 mol/L HEPES, pH 7.4 and 9% (*w*/*v*) polyethylene glycol 3350 at 18°C by the microbatch-under-oil method. The binary complex with ssRNA (5′-AGAUCAA-3′) was also obtained through cocrystallization. Initially, ZIKV helicase_172–617_ (storage buffer: buffer D) at 5 mg/mL was incubated with ssRNA (GenePharma) at 0.2 mmol/L (~2-fold molar excess) at 18°C for 1 h. Subsequently, the crystals of ZIKV helicase_172–617_-RNA complex were grown at 18°C by the microbatch-under-oil method and the crystallization condition contained 0.2 mol/L potassium sodium tartrate tetrahydrate pH 7.4 and 20% (*w*/*v*) polyethylene glycol 3350.

### Crystal data collection, structure determination and refinement

Crystals were cryoprotected using the crystallization buffer with 30% glycerol and flash-frozen in liquid nitrogen. Diffraction data were collected at 100 K at Shanghai Synchrotron Radiation Facility (SSRF) beamLine BL19U1 at a wavelength of 0.97853 Å. Diffraction data were processed using HKL3000 (Minor and Otwinowski, 1997). Crystals for both complexes belong to space group *P2*_1_ and the data statistics are summarized in Table S1. The structures were solved by molecular replacement using the apo structure of ZIKV helicase (PDB ID 5JMT) as a search model. The program PHASER (McCoy et al., 2007) was used for the molecular replacement search. The initial models were auto-built by Buccaneer (Cowtan, 2006) and refined through iterative rounds of TLS and restrained refinement using Refmac5 (Murshudov et al., 2011), followed by rebuilding manually using Coot (Emsley and Cowtan, 2004). The refinement statistics are summarized in Table S1.

### Protein structure accession number

The refined coordinates have been deposited in the PDB under accession number 5GJB and 5GJC.

## Electronic supplementary material

Below is the link to the electronic supplementary material.

Supplementary material 1 (PDF 171 kb)
