# Supplementary material for: Structural basis of Zika virus helicase in recognizing its substrates
Source: Protein Cell. 2016 Jul 18;7(8):562–70. doi: 10.1007/s13238-016-0293-2 (PMC4980333; doi:10.1007/s13238-016-0293-2)
Supplement: Supplementary file 1 — Supplementary material 1 (PDF 171 kb) [file 13238_2016_293_MOESM1_ESM.pdf]

SUPPLEMENTAL MATERIALS

Supplementary Table S1. Data collection, phasing and refinement statistics

	ZIKV helicase-ATP-Mn^{2+}	ZIKV helicase-RNA
**Data Collection**		
X-ray Source	SSRF beamline BL19U1	
Wavelength (Å)	0.97853 Å	
Space group	$P2_1$	
Unit cell a, b, c (Å)	51.6, 74.2, 57.7,	52.3, 72.3, 59.4,
α, β, γ (°)	90.0, 92.2, 90.0	90.0, 94.5, 90.0
Resolution range* (Å)	40.0-2.20 (2.24-2.20)	50.0-1.70 (1.73-1.70)
Unique reflections	21839 (1095)	39662 (780)
Completeness (%)	99.3 (99.5)	82.0 (32.3)**
Redundancy	6.7 (6.6)	6.6 (5.2)
$I/\sigma I$	22.8 (2.0)	14.9 (2.9)
R_{merge}	0.083 (0.942)	0.130 (0.506)
**Refinement**		
Resolution range (Å)	37.7-2.20	40.7-1.70
No. of reflections (working/test)	40680/2043	73821/4142
R_{work}/R_{free}	0.1970/0.2474	0.1539/0.1956
Number of atoms		
Protein	3396	3479
Water	107	549
Ligand/ion	32	104
B-factors		
Protein	55.1	18.6
Water	51.9	29.6
Ligand/ion	82.7	16.7
r.m.s. deviations		
Bond lengths (Å)	0.004	0.008
Bond angles (°)	0.914	1.145

* Numbers in the brackets are for the highest resolution shell.

**Completeness is over 90% at resolution shell of 2.02 Å-1.96 Å.
